# The heterogeneous regional effect of mobility on Coronavirus spread

**DOI:** 10.1140/epjs/s11734-022-00533-6

**Published:** 2022-03-20

**Authors:** José Manuel Amoedo, Yago Atrio-Lema, María del Carmen Sánchez-Carreira, Isabel Neira

**Affiliations:** 1 Department of Applied Economics, ICEDE Research Group, Faculty of Economics, Universidade de Santiago de Compostela, Santiago de Compostela, Spain; 2 Department of Quantitative Economics, VALFINAP Research Group, Faculty of Economics, Universidade de Santiago de Compostela, Santiago de Compostela, Spain; 3 Department of Applied Economics, ICEDE Research Group, Faculty of Economics, CRETUS, Universidade de Santiago de Compostela, Santiago de Compostela, Spain

## Abstract

The Coronavirus (COVID-19) pandemic struck global society in 2020. The pandemic required the adoption of public policies to control spread of the virus, underlining the mobility restrictions. Several studies show that these measures have been effective. Within the topic of Coronavirus spread, this original paper analyses the effect of mobility on Coronavirus spread in a heterogeneous regional context. A multiple dynamic regression model is used to control sub-national disparities in the effect of mobility on the spread of the Coronavirus, as well as to measure it at the context of Spanish regions. The model includes other relevant explanatory factors, such as wind speed, sunshine hours, vaccinated population and social awareness. It also develops a new methodology to optimise the use of Google trends data. The results reveal heterogeneity among regions, which has important implications for current and future pandemic containment strategies.

## Introduction

On 11 March 2020, the World Health Organization (WHO) declared the emergency caused by the outbreak of a new Coronavirus (COVID-19) to be an international pandemic. Previously, Asian countries such as China (particularly the Wuhan region), South Korea and Singapore had been severely affected by the Coronavirus, necessitating actions to contain its spread. By early March 2020, Italy (with the outbreak in Lombardy) was the only European country that had applied restrictions. Subsequently, most Western countries applied more severe restrictions to contain spread. Spain, France, Germany, the UK, the USA, and many others applied new restrictions, the strictest being home confinements. These policies were extended throughout 2020 and 2021, with continuous changes due to the different waves and being reduced gradually as vaccination has progressed until the last months of 2021. In addition, restrictions have varied over time, including perimeter closures, time restrictions, capacity reductions or closure of certain areas.

The aim of these policies was to increase social distancing and reduce the personal contact responsible for Coronavirus spread [1, 2]. One consequence of this policy is the reduction in economy activity as is closely related to the spread of viral diseases (among them, Coronavirus) and to human mobility [2–5].

Various authors have analysed the mobility restrictions that most Western governments used to contain the pandemic. Studies follow three different lines that are closely related but use different methodologies and measures. The first line examines the effects of mobility restrictions on evolution of Coronavirus spread, focussing on lockdown policies [2, 4, 6].^1^ Such papers perform a two-step analysis, first examining the efficacy of mobility restrictions by measuring the changes in human mobility and then analysing the correlation between mobility and Coronavirus spread. The second line analyses the effect of mobility on Coronavirus spread without specifically evaluating the restrictions [1, 7–9]. Other works pursue questions closely related to mobility, such as transport accessibility [10].

Socioeconomic, physical or biological studies of the factors that determine spread of the illness have focussed on analysing individual countries [1, 10–13], groups of countries [2, 14–17], and to a lesser extent regions and cities [6–9]. The literature has shown the effect of specific factors (mobility, weather, vaccination process, social awareness, lockdown of areas with high concentrations of people or transport accessibility, among others) in specific geographic areas by analysing the average effect of each factor in each region. However, socioeconomic studies of regional scope should consider the possibility of heterogeneous behaviour in different regions in the presence of certain factors, as occurs with mobility restrictions.

In this way, the novelty of this paper is to control sub-national disparities in the effect of mobility on the spread of the Coronavirus, as well as to measure it. Moreover, it includes the variable of vaccinated people in the analysis.

The study focuses on Spanish regions for three main reasons: the high incidence of COVID-19 in Spain, the high level of decentralisation and thus regional heterogeneity,^2^ and the high level of vaccination. Such knowledge is very important to adapting national and regional government actions to the reality of each territory.

The factors that explain the spread of Coronavirus can be grouped into the following four groups: mobility, deeply related to social distancing; social awareness, environmental aspects and other factors.

Concerning the first group of factors, different authors have analysed the impact of human mobility and economic activity on the spread of viral diseases before the Coronavirus pandemic [3, 18] and in its wake [4, 12, 19]. Using datasets from France, Adda [3] observes a positive relationship between interregional trade and faster spread of a viral disease. The study shows how the globalisation process (expansion of transportation networks and growth in trade) can explain part of the rise in transmission rate of viral diseases in the last quarter of the twentieth century. Epstein et al. [18] focus on restrictions on passenger flights and the spread of flu in the United States, showing how flight restrictions, combined with other restrictions, slow spread but at an economic cost. Analysing the effect of mobility on Coronavirus spread, Kuo and Fu [12] demonstrate that reopening business activities accelerated spread. Another interesting question, studied by Adda [3] and Kuo and Fu [12] involves the need for restrictions to contain the spread of viral diseases. These restrictions are closely related to the economy and the human mobility. Thus, the trade-off between public health and economic activity should be considered. Most restrictions limit mobility (people and trade) and personal contact (social distancing), closing public spaces and institutions with high concentrations of people (e.g., schools, sport stadiums, cultural events) or closing shops and businesses [2, 12].

The main conclusion of previous research on this topic is the need for public action, involving mobility restrictions, to contain the spread of any viral disease that constitutes a special potential danger for society. A few studies of mobility restrictions and lockdowns in different parts of the world support this conclusion in the case of the Coronavirus [1, 2, 4, 6, 9, 12, 19]. A considerable number of them analyse countries and regions strongly affected by the pandemic in its initial stages. China (specifically Wuhan) and Italy have received the most attention. Other researches reach the same conclusion using international datasets [2, 20]. Nevertheless, imposing strict mobility constraints on movement of people and freight within and beyond national boundaries in the modern and global era is detrimental to the economy and to business development [2].

With respect to the first country affected, China, various works on issues such as the Wuhan lockdown reach essentially the same conclusion. Mobility restrictions are effective if the key aim is to contain Coronavirus spread^3^ [6, 9]. Other papers stress the effect of mobility on the spatial and temporal spread of Coronavirus, showing how mobility generates different numbers of infections and deaths at different stages of the pandemic [8].

Research of Italy and China reach very similar conclusions. Mobility plays a central role in the spread of Coronavirus in Italy’s regions, as do other factors, such as temperature, air pollution and nearness to outbreak points [1]. Other authors consider transport accessibility as the most significant factor explaining the Coronavirus pandemic [10]. In parallel, other works concur on the importance of mass transport in Coronavirus spread [2] and the need to reduce its use through stay-at-home policies [21]. Many of these aspects will be discussed below and some of them will be included in the empirical analysis.

As abovementioned, the Coronavirus pandemic has had a global impact, but it has struck some countries harder than others. Although China was the first country affected, it was not ultimately the hardest hit. The US, the UK, Italy, and Spain were the countries most affected during the first stage. Later, other countries as Brazil or India were harder hit. The greater severity of the pandemic in these countries can be explained by social and institutional factors, such as a higher proportion of elderly people, employment in the service sector and globalisation [2] and the differences in the time can be explained by the climatic seasons, among others. This study also shows the positive effect of mobility restrictions. It finds that reducing mobility in transit stations (TS), retail and recreation facilities (RR) and workplaces (WP) reduces^4^ pandemic severity^5^ (PS). Conversely, increased mobility in residences (RD) leads to a reduction in pandemic severity.

The analysis of the datasets of five large cities in the US shows striking differences among cities in the effect of mobility reduction on the decline in cases per capita. New York City is the most significant case, and lockdown policies again play an important role [7]. Bonaccorsi et al. [22] analyse the effects of mobility restrictions on economic activity (which is closely connected to human mobility) in Italian cities but neither establish nor measure the effect of these restrictions on spread of the virus. Cartenì et al. [1] use mobility among Italian regions but obtain an average effect for the national government. In sum, the regional analyses in the mentioned studies do not consider the possible heterogeneity of regions or measure the impact of mobility in each region.

There are two reasons for these omissions. The first reason involves both the use of cities, a different concept than the region, and the use of different measures of mobility and variables for each city. This approach prevents researchers from isolating the effect of mobility. The second reason is not measuring the impact of mobility on Coronavirus spread in each region. This occurs because the study does not establish the relationship but only the impact of mobility restrictions or calculates the average impact for all regions.

Regarding the second groups of factors, Milani [15] and Tiwari et al. [23] identify social awareness as another important factor in Coronavirus spread. As the pandemic advanced, public attention and fear of Coronavirus changed. Social awareness is significant in the fight against Coronavirus spread. Prior studies, such as Geoffard and Philipson [24], argue the need for social awareness in the fight against Human Immunodeficiency Virus (HIV). A higher social awareness of the pandemic personal and social consequences is usually associated with greater self-protection and respect for third parties safety. Such awareness impacts pandemic severity negatively, reducing new cases and deaths. Several recent works have analysed this relationship in different countries and regions using Google Trends datasets with different terms and topics [14, 15, 25].

Google Trends enables measurement of the population risk perception by selecting any terms closely related to the pandemic. This indicator of social awareness may be more accurate than official new cases or deaths, which are an imperfect measure [15]. Fantazzini [14] analyses risk perception in 158 countries, showing that this indicator provides useful information for measuring social awareness using the topics “Coronavirus” and “pneumonic” as health categories.

Most existing research examines the impact of fear on the stock market and financial economy. This research demonstrates that an increase in Google searches with different terms related to COVID-19 (e.g. “Coronavirus”, “corona”, “World Health Organization”, “virus”, “COVID-19”, “Symptom”, “unemployment”, “laid off”) is significantly and negatively associated with variations in stock market prices [26–28]. It also uses Google Trends data to measure other issues, such as the level of self-protection against the virus [25] and public attention. These issues are closely related to communications from public health authorities [29].

A common and relevant aspect in the literature discussed in the preceding paragraphs is the study of early phases of the pandemic. In these initial phases, the lack of knowledge about the Coronavirus was remarkable, thus, the effects of Social Awareness should become less important over time and even disappear.

With respect to the third group of factors, several studies analyse weather conditions, such as temperature [30], humidity or precipitations [30–33], daylights hours [31–34], wind speed [31, 32, 35] or air pollution [1]. The weather conditions are deeply interrelated. Specifically, high temperature and low relative humidity generate a significant reduction of virus spread. The second factor is more relevant, because the droplet cloud travelled distance and concentration remain significant at any temperature if the relative humidity keeps high [30, 31]. There is an exception to this, since the aerosol particles increase in high temperature and low humidity environments leading to the Coronavirus spread [32].

Other works include sunshine hours as an alternative factor to explain the Coronavirus spread [31, 34]. More sunshine hours increase the number of new cases and deaths [31]. Sagripanti and Lytle [34] indicate that Coronavirus aerosolised form infected patients and deposited on outdoors surfaces may remain infectious for a considerable time during the winter in many temperate zones. Specifically, 90% of Coronavirus particles are inactivated after 11–34 min of exposure to sunlight in most parts of the world during the summer. In contrast, the virus will persist infectious for at least one day in winter.

The last analysed factor is the wind speed, which increases the number of cases and deaths [31]. The improper airflow and higher wind velocity can strongly increase the travelling distance of aerosol particles and droplets, the needed social distancing, and the risk of Coronavirus transmission [32, 35]. A limitation to the increase of virus spread generated by the wind is the wearing face coverings (generally masks). Chea et al. [35] analyse the use of mask (specifically, N95 mask) in the Coronavirus spread, finding It highly effective.

Concerning the last group of factors, some studies analyse issues that are related to society and institutions, diagnostic capability, or vaccination process. Concerning socioeconomic and institutional factors related to Coronavirus spread, population density [1, 2], elderly people, globalisation level, employment in the service and agricultural sector, and education level [2] are common. Other relevant factor is the number of swabs performed, which is an important control variable [1], because it is closely related to diagnosis of Coronavirus.

Finally, the vaccination process, has been examined to a lesser extent due to its recent onset. Previously to the start of the vaccination process, two main groups of studies can be identified. First, some authors study issues, such as the herd effect, showing the risk inherent to the strategies that seek it as main goal [36] or the necessary requirements to achieve this [37]. Second, other works analyse the effect of the population ratio vaccinated for other diseases (used to similar respiratory viruses) in different countries, showing the significative reduction in Coronavirus spread [38]. Recently, different papers directly study the effect of vaccination process in the Coronavirus spread and mortality. These studies show the reduction in Coronavirus spread and mortality generated by the vaccination process (increase of vaccinated population with one or two doses) due to the increase of immunisation. Some works highlight the need to combine the vaccination process and the mobility restrictions to avoid the Coronavirus spread [39, 40].

The literature review suggests the significance of various factors in Coronavirus spread. After analysing these factors, empirical analyses were performed to test their incidence in Spain from a regional perspective [39, 40].

## Materials and methods

### Materials

As stated above, one aim of this paper is to investigate the influence of mobility restrictions on Coronavirus (COVID-19) spread during the pandemic. The unit of regional analysis was NUTS 2 for Spain. The data used in the estimates were collected from the following sources:

- Reported cases of COVID-19 per 1,000,000 inhabitants between 15 March 2020 and 15 November 2021 [41]. Sample selection ranges from April^6^ to November 2021 and it is described in Appendix A.1, Table 3.^7^
- Mobility habits collected between 15 March 2020 and 15 November 2021 [42] (described in Appendix A.1, Table 4).
- Search interest of the term “Coronavirus” as measured by Google searches [43] between 15 March 2020 and 15 November 2021^8^ (described in Appendix A.1, Table 5).
- Sunshine hours in a day [44] collected between 15 March 2020 and 15 November 2021 (described in Appendix A.1, Table 6).
- Wind speed (daily average) [44] between 15 March 2020 and 15 November 2021 (described in Appendix A.1, Table 7).
- Percentage of the population vaccinated [45] between 15 March 2020 and 15 November 2021 (described in Appendix A.1, Table 8).

A pool data with 10353 observations and 17 regions during a period of just over 22 months was created using daily data. The pooled data provided a more complete database and enabled control of individual heterogeneity and more accurate identification of the adjustment dynamics.

- Dependent variable:

**Table 1:** Effect of mobility habits on Coronavirus spread by region

Dependent variable	Coronavirus spread (mean = 168.01)	4-23-2020 to 11-15-2021
Model	Least squares (1)	Panel GMM (2)
Variables	Coefficient (std. error)	Coefficient (std. error)
Mobility habits (− 14)	2.474 (0.216)***	3.130 (0.276)***
Mobility habits (− 14) $\times$ Aragon	0.810 (0.149)***	1.324 (0.163)***
Mobility habits (− 14) $\times$ Asturias	0.189 (0.079)**	− 0.201 (0.109)*
Mobility habits (− 14) $\times$ Balearic Islands	0.117 (0.060)*	0.196 (0.079)**
Mobility habits (− 14) $\times$ Valencian Community	0.044 (0.070)	0.164 (0.082)**
Mobility habits (− 14) $\times$ Canary Islands	− 0.374 (0.117)***	− 0.840 (0.142)***
Mobility habits (− 14) $\times$ Cantabria	− 0.281 (0.074)***	− 0.290 (0.100)***
Mobility habits (− 14) $\times$ Castile-La Mancha	1.330 (0.167)***	1.953 (0.132)***
Mobility habits (− 14) $\times$ Castile and Leon	0.903 (0.145)***	1.393 (0.161)***
Mobility habits (− 14) $\times$ Catalonia	0.264 (0.123)**	0.626 (0.132)***
Mobility habits (− 14) $\times$ Extremadura	1.067 (0.125)***	1.515 (0.157)***
Mobility habits (− 14) $\times$ Galicia	− 0.166 (0.064)***	− 0.238 (0.098)**
Mobility habits (− 14) $\times$ La Rioja	0.795 (0.135)***	1.206 (0.152)***
Mobility habits (− 14) $\times$ Madrid	0.473 (0.123)***	0.850 (0.131)***
Mobility habits (− 14) $\times$ Murcia	0.665 (0.089)***	0.967 (0.109)***
Mobility habits (− 14) $\times$ Navarre	0.429 (0.137)***	0.864 (0.139)***
Mobility habits (− 14) $\times$ Basque Country	0.046 (0.120)	0.291 (0.126)**
Social awareness (− 10)	0.146 (0.221)	− 0.129 (0.341)
Coronavirus spread (− 1)	0.889 (0.018)***	0.812 (0.020)***
Sun (14)	− 0.929 (0.224)***	− 0.815 (0.245)***
Wind speed (14)	− 1.355 (0.704)*	0.262 (0.712)
Vaccinated (− 44)	− 0.361 (0.059)***	− 0.524 (0.071)***
Adjusted *R*-squared	0.8307	0.8250
DW statistic	2.057	1.839
*N*	9605 ($t=565$, $i=17$)	9605 ($t=565$, $i=17$)

Source: The authors

Signif: ***0.01, **0.05, *0.1

Coronavirus spread: measured by reported cases of COVID-19 per 1,000,000 inhabitants.

- Explanatory variables

Main objective: habitual mobility^9^ was used to study mobility habits. This variable represents the percentage of population that leaves its area of residence^10^ during working hours in each region of Spain. It serves as a proxy for variation in labour mobility and is based on aggregate data (total origin-destination flows) [46].

- Other control variables:

Social awareness: captures the search interest of the term “Coronavirus” throughout the analysed period. Google Trends provides a standardised time series, in which the day with the highest relative number of searches takes the value of 100. The regional values are also standardised, assigning the value 100 to the region with the highest relative volume of searches throughout the period. One limitation is that Google Trends API provides weekly or monthly rather than daily data for periods longer than 3 months. This limitation makes it impossible to obtain daily panel data for series of longer than 3 months, greatly reducing the utility of this data source.

In view of the foregoing, this paper develops a new methodology to overcome this limitation by combining three processes. First, quarterly regional data are panelised using Coello [47] repository. Second, the data are connected by a simple chain index [48]. Finally, the data are standardised using the procedure proposed by Narita and Yin [49] (see Appendix A.2 for more information).

It means a relevant methodological contribution, which provide valuable tools for future studies that use Google Trends as an information source. The process developed here to obtain a daily data pool for periods longer than 3 months enables the use of Google searches in many fields of knowledge for long periods, extending the study by Narita and Yin [49].

- Sun: measured as the average of the sunlight hours of all the meteorological stations collected in AEMET [44] for each of the regions.
- Wind speed: measured as the average of the wind speed of all meteorological stations collected in AEMET [44] for each of the regions.
- Vaccination: measured by the percentage of vaccinated population [45].
- Region: dummy variable for each of the 17 analysed regions.

### Methods

The goal of this study is to estimate the effect of mobility on spread of the Coronavirus in a geographical area of regional scope. Thus, the study aims to examine, not only to control, the magnitude of possible regional heterogeneity. This goal requires using parametric estimation. Among the feasible alternatives, linear parametric estimation is the most appropriate because it is easy to understand and interpret, and because it fits more complex nonlinear specifications well.

The analysis starts from a pooled specification:

1 $$\begin{aligned}&{\text {Coronavirus spread}}_\mathrm{it}\nonumber \\&\quad =\alpha _{i}\mathrm{Region}_{i}\mathrm{MobilityHabits}_\mathrm{it}+\sum \limits _{j=0}^4 \beta _{j}x_{{j}_{it}} +\varepsilon _\mathrm{it}\nonumber \\ \end{aligned}$$

where subscripts *i* refers to the region ($i=1,\ldots ,17$), *t* to the day ($t=1,\ldots , 610$) and *j* to the control variables ($j=1,2,3,4$). Least Squares (LS) procedure is then performed. To confirm the appropriateness of the proposed equation, one must first contrast the hypothesis of heterogeneous effects of mobility on spread of Coronavirus among regions. This contrast is performed using the Wald Test [50] to determine whether homogeneity exists between parameters $\alpha _{{i}}$. The null hypothesis is rejected, affirming that the effects of mobility differ among regions.

Since the lagged dependent variable is correlated with the errors, even if there are no autocorrelation problems, the estimators used in static models would be inconsistent. Therefore, it would be necessary to resort to the use of estimators based on the Panel Generalized Method of Moment (GMM) [51].^11^

Because this is a daily sample, the Durbin Watson Test is used to detect possible self-correlation problems.^12^ The result of the alternative estimation is the Panel Generalized Method of Moment^13^ (GMM) presented in column (2) of Table 1.

## Results

The results of estimating Eq. (1) using different methods are presented in Table 1.

The variable mobility habits was estimated independently for each region, and globally for all the regions (variables Mobility Habits), in both models to obtain each regional effect. Thus, the estimated coefficients for each region must be added to the overall effect. To avoid multicollinearity, one region (specifically Andalusia) has been omitted, with its estimated effect corresponding to that of the global variable. The other variables were estimated jointly for all regions.

**Fig. 1:**
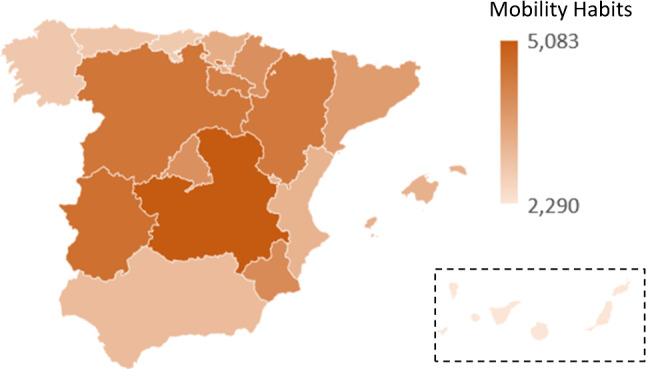
Regionas effects Source: The authors

In both estimations, mobility habits (habitual mobility) is positively related (in most regions) to Coronavirus spread after 14 days. For example, in Aragon, an increase in mobility habits of one percentage point (14.49%, mean Appendix A.1, Table 4) induces an increase (statistically significant) of 4.454 cases^14^ per 1,000,000 inhabitants (mean of 100 in the analysed period) in Coronavirus spread 14 days later. In other words, if mobility habits decrease by one percentage point in Andalusia, the reported cases of COVID-19 in Andalusia can be expected to decrease by 6 people (over the total population) (all regional data are presented in Appendix A.1, Table 8). These results are similar to the findings of Cartenì et al. [1]. One major conclusion from the estimated coefficients of the effect of mobility habits on Coronavirus spread is the regional heterogeneity of these effects. Map 1 presents this regional heterogeneity on mobility. The effects in the regions of Cantabrian coast (northern and north-western Iberian Peninsula, including Galicia (2892), Asturias (2929) and Cantabria (2840) are clearly smaller than in the centre and northeast of Spain. The latter do, however, show striking differences. Castile-La Mancha (5083), especially, Extremadura (4645), Castile and Leon (4523), Aragon (4454) and La Rioja (4336) have different and worse performance than the other regions. Below these values, but clearly ahead of the other regions (situated between 3 and 4.1), Murcia (4097), Navarre (3994), Madrid (3980), Catalonia (3756) and Basque Country (3421). Andalusia (3130), the Valencian Community (3294) and the Basque Country (3421) present similar values to the Cantabrian coast, but slightly higher. The two archipelagos (Canary and Balearic Islands) have similar situations (2290 and 3326, respectively), with very low effects of mobility, mainly on the first one (Fig. 1).

If social awareness increases by one percentage point, the severity of the pandemic decreases by 0.129 cases (in dynamic estimation) 10 days later. These values establish a inverse relationship, as expected and in line with other studies [15] but not significant. This could be due to the loss of effect over an extended period such as the one under discussion.

An increase in Sun of one daily sunlight hour induces a decline (statistically significant) of 0.815 cases per 1,000,000 inhabitants 14 days later. If wind speed increases 1 km/h the severity of the pandemic decreases by 0.262 cases 1,000,000 inhabitants 14 days later, being this not significant.

If population vaccinated increases by one percentage point, the new cases 44^15^ days later decrease significantly in 0.524 cases per 1,000,000 inhabitants

## Conclusions

**Table 2:** Reported cases of COVID-19 per 1,000,000 inhabitants: descriptive statistics

Region	Mean	SD	Skewness	Kurtosis	Min	Max
Andalusia	157.35	181.58	1.91	3.90	0.00	1041.57
Aragon	202.62	194.22	1.52	2.07	0.00	972.49
Asturias	115.18	138.03	1.80	3.04	0.00	789.99
Balearic Islands	145.64	184.68	2.04	4.16	0.00	986.55
Valencian Community	169.35	286.36	3.20	11.73	0.00	1928.75
Canary Islands	74.45	74.60	2.28	6.87	0.00	455.10
Cantabria	133.18	126.15	1.40	1.92	0.00	726.24
Castile-La Mancha	194.64	231.38	2.68	9.73	0.00	1547.08
Castile and Leon	198.85	237.57	2.02	4.11	0.00	1273.99
Catalonia	198.99	216.18	2.31	6.16	0.00	1322.18
Extremadura	157.91	219.63	2.64	8.32	0.00	1367.41
Galicia	114.88	137.34	2.02	4.03	0.00	736.43
La Rioja	205.28	229.32	1.96	4.76	0.00	1379.43
Madrid	221.21	233.02	1.55	2.11	0.00	1167.12
Murcia	157.57	231.80	2.89	10.22	0.00	1530.22
Navarre	211.27	209.28	1.64	2.54	0.00	1125.01
Basque Country	197.76	159.67	1.04	0.79	1.81	778.16

Source: The authors

This paper analyses the heterogeneous spread of Coronavirus in Spanish regions. To study the relationship between mobility and Coronavirus spread, mobility was measured by number of people who leave their geographic area, understood as the surrounding area inhabited by 5000 people. This mobility is closely related to the lockdowns decreed by Spanish regions at different stages of the pandemic, measures that attempt to contain the pandemic and reduce cases. This research found that these measures do not have the same effect in all regions, as the relationships between nearby areas differ greatly.

The analysis shows the heterogeneous effect of Coronavirus on Spanish regions. This paper confirms that daily new Coronavirus cases are directly related to mobility habits 14 days before. Other issues, such as social awareness, weather factors and vaccinated population, are also relevant in explaining Coronavirus spread. This paper also considers the percentage of vaccinated people as a relevant factor in explaining the Coronavirus spread. The inclusion of this key aspect means a step beyond previous studies. Specifically, the high significance of the percentage of vaccinated people in reducing the number of new infections is confirmed. It should be noted that Spain is among the countries with the highest vaccination rates.

**Table 3:** Mobility habits: descriptive statistics (%)

Region	Mean	SD	Skewness	Kurtosis	Min	Max
Andalusia	14.38	3.00	$-$ 0.64	0.12	5.17	20.11
Aragon	14.49	3.29	$-$ 0.27	$-$ 0.17	5.21	22.17
Asturias	14.82	3.78	$-$ 0.16	$-$ 0.32	5.24	24.28
Balearic Islands	15.46	3.81	$-$ 0.78	0.23	3.92	22.97
Valencian Community	16.85	3.64	$-$ 0.69	0.25	5.88	23.84
Canary Islands	15.45	3.37	$-$ 0.43	0.11	5.17	24.80
Cantabria	16.03	4.00	$-$ 0.18	$-$ 0.43	5.84	25.78
Castile-La Mancha	11.88	2.71	$-$ 0.61	0.13	3.96	17.21
Castile and Leon	13.60	2.95	$-$ 0.43	$-$ 0.07	5.15	20.14
Catalonia	15.84	3.66	$-$ 0.31	$-$ 0.23	6.02	24.26
Extremadura	16.85	3.64	$-$ 0.69	0.25	5.88	23.84
Galicia	11.97	2.50	$-$ 0.60	0.03	4.59	17.04
La Rioja	15.44	3.63	$-$ 0.39	$-$ 0.40	5.97	22.91
Madrid	16.22	4.04	$-$ 0.19	$-$ 0.50	5.95	24.59
Murcia	13.39	2.85	$-$ 0.21	$-$ 0.09	5.13	20.19
Navarre	15.44	3.47	$-$ 0.41	$-$ 0.05	5.33	23.29
Basque Country	17.25	4.13	$-$ 0.29	$-$ 0.40	6.45	26.15

Source: The authors

**Table 4:** Search interest of the term “Coronavirus”: descriptive statistics

Region	Mean	SD	Skewness	Kurtosis	Min	Max
Andalusia	6.39	9.82	3.57	14.53	0.24	76.19
Aragon	7.50	7.53	3.63	15.48	0.47	62.79
Asturias	7.80	9.81	3.49	13.58	0.00	75.39
Balearic Islands	5.43	9.99	3.56	14.71	0.00	77.88
Valencian Community	6.02	7.95	3.63	16.15	0.15	66.77
Canary Islands	5.39	8.98	3.31	11.83	0.02	59.93
Cantabria	7.32	5.73	2.90	11.45	0.00	43.80
Castile-La Mancha	6.35	10.25	3.51	13.94	0.15	79.64
Castile and Leon	6.98	9.32	3.59	15.31	0.21	76.77
Catalonia	4.84	7.87	3.66	15.32	0.12	60.74
Extremadura	6.02	7.95	3.63	16.15	0.15	66.77
Galicia	7.59	7.58	3.61	15.78	0.00	65.29
La Rioja	6.29	10.09	3.70	15.27	0.28	73.60
Madrid	5.96	8.52	3.52	14.24	0.45	67.45
Murcia	6.36	7.29	3.44	14.21	0.00	55.44
Navarre	8.57	10.01	3.37	13.91	0.00	81.97
Basque Country	7.11	7.89	3.34	13.47	0.06	62.21

Source: The authors

**Table 5:** Wind speed: descriptive statics

Region	Mean	SD	Skewness	Kurtosis	Min	Max
Andalusia	2.94	0.82	0.85	1.00	1.31	6.39
Aragon	2.49	0.89	0.84	0.36	0.85	5.34
Asturias	2.94	1.02	1.46	2.91	1.30	8.12
Balearic Islands	3.42	1.10	1.26	3.06	1.36	10.30
Valencian Community	2.72	0.78	1.44	4.04	0.94	6.64
Canary Islands	4.62	1.11	0.12	$-$ 0.43	2.15	8.45
Cantabria	3.09	1.28	1.28	1.91	0.78	8.53
Castile-La Mancha	2.51	0.92	1.08	1.69	0.93	6.41
Castile and Leon	2.83	0.97	1.01	1.19	1.14	6.68
Catalonia	2.54	0.59	0.79	1.61	1.30	5.31
Extremadura	2.61	0.96	1.04	1.29	0.99	6.50
Galicia	3.22	1.10	1.08	1.39	1.10	7.74
La Rioja	2.99	1.43	1.36	2.71	0.30	9.40
Madrid	2.86	1.13	1.08	1.73	0.80	7.88
Murcia	2.62	0.72	1.04	2.08	0.94	6.50
Navarre	2.89	1.32	0.50	$-$ 0.35	0.30	6.95
Basque Country	3.02	1.08	1.08	1.21	1.12	7.92

Source: The authors

**Table 6:** Sun: descriptive statics

Region	Mean	SD	Skewness	Kurtosis	Min	Max
Andalusia	8.20	3.16	$-$ 0.63	$-$ 0.53	0.04	13.21
Aragon	7.59	3.43	$-$ 0.40	$-$ 0.76	0.00	13.13
Asturias	5.33	3.66	0.34	$-$ 0.99	0.00	13.75
Balearic Islands	8.07	3.54	$-$ 0.43	$-$ 0.71	0.00	13.68
Valencian Community	8.30	3.45	$-$ 0.70	$-$ 0.26	0.00	13.59
Canary Islands	8.38	2.26	$-$ 0.72	0.33	0.21	12.32
Cantabria	5.77	4.16	0.24	$-$ 1.08	0.00	14.43
Castile-La Mancha	7.75	3.26	$-$ 0.70	$-$ 0.57	0.00	12.02
Castile and Leon	7.28	3.68	$-$ 0.13	$-$ 1.16	0.00	13.46
Catalonia	7.50	3.40	$-$ 0.42	$-$ 0.65	0.00	13.36
Extremadura	8.66	3.89	$-$ 0.64	$-$ 0.74	0.00	13.81
Galicia	6.37	3.78	0.02	$-$ 1.17	0.00	13.55
La Rioja	7.27	4.37	$-$ 0.19	$-$ 1.14	0.00	14.40
Madrid	7.92	4.09	$-$ 0.45	$-$ 0.92	0.00	13.97
Murcia	8.53	3.42	$-$ 0.70	$-$ 0.18	0.00	13.53
Navarre	6.37	4.33	0.07	$-$ 1.27	0.00	14.40
Basque Country	5.41	3.84	0.28	$-$ 1.09	0.00	13.70

Source: The authors

**Table 7:** Percentage of vaccinated: descriptive statics

Region	Mean	SD	Skewness	Kurtosis	Min	Max
Andalusia	17.68	27.95	1.33	0.09	0.00	79.74
Aragon	37.84	27.31	0.11	$-$ 1.46	0.00	79.47
Asturias	39.93	28.57	0.16	$-$ 1.39	0.00	85.36
Balearic Islands	36.18	27.03	0.09	$-$ 1.48	0.00	85.07
Valencian Community	36.08	27.14	0.19	$-$ 1.39	0.00	79.85
Canary Islands	33.10	25.12	0.20	$-$ 1.42	0.00	73.31
Cantabria	36.22	26.70	0.23	$-$ 1.31	0.00	82.34
Castile-La Mancha	37.50	27.20	0.11	$-$ 1.47	0.00	82.11
Castile and Leon	39.65	28.07	0.10	$-$ 1.44	0.00	82.39
Catalonia	35.91	26.53	0.13	$-$ 1.47	0.00	76.80
Extremadura	38.92	28.25	0.16	$-$ 1.41	0.00	83.65
Galicia	40.09	29.15	0.10	$-$ 1.44	0.00	84.95
La Rioja	38.32	27.75	0.12	$-$ 1.43	0.00	81.35
Madrid	37.76	27.94	0.11	$-$ 1.48	0.00	84.66
Murcia	35.77	26.61	0.17	$-$ 1.43	0.00	77.64
Navarre	37.11	27.09	0.14	$-$ 1.43	0.00	79.57
Basque Country	37.95	28.04	0.12	$-$ 1.44	0.00	81.63

Source: The authors

**Table 8:** Mobility habits: effects by region

Region	Mobility habits	Population	Coronavirus spread
Andalusia	3.13	8,414,240	26
Aragon	4.45	1,319,291	6
Asturias	2.93	1,022,800	3
Balearic Islands	3.33	1,149,460	4
Valencian Community	3.29	5,003,769	16
Canary Islands	2.29	2,153,389	5
Cantabria	2.84	581,078	2
Castile-La Mancha	5.08	2,032,863	10
Castile and Leon	4.52	2,399,548	11
Catalonia	3.76	7,675,217	29
Extremadura	4.64	1,067,710	5
Galicia	2,89	2,699,499	8
La Rioja	4.34	316,798	1
Madrid	3.98	6,663,394	27
Murcia	4.10	1,493,898	6
Navarre	3.99	654,214	3
Basque Country	3.42	2,207,776	8

Source: The authors

The policy implications of these heterogeneous effects suggest applying different measures at regional level. Then, establishing mobility restrictions in the NUTS of central Spain would have a stronger effect. During the first stage of restrictions (March–June 2020), the same measures were applied throughout Spain. Afterwards, different levels of de-escalation were applied based on other criteria, such as population size. Later, other restrictive policies (such as time restrictions, capacity restrictions or geographic closures) were applied with the same objective. Subsequently, the measures were primarily decided and applied at the regional level, allowing governments to tailor their policies better to regional specificities.

The main limitations of this research involve the availability and quality of the data. More granular regional data were needed for mobility areas.

Finally, analysing the implications of heterogeneous context for restrictive policies at different regional and local levels requires further investigation. Future research could also extend this study to develop new modelling that includes more territorial variables to control for spatial heterogeneity.

## Appendix A

### Appendix A.1

See Tables 2, 3, 4, 5, 6, 7 and 8.

### Appendix A.2

*Illustration 1. Panelised volume index*

$$\begin{aligned} X= \left( \begin{array}{llllll} x_{11} &{}\quad x_{12} &{}\quad \ldots &{}\quad x_{1j} &{}\quad \ldots &{}\quad x_{1t}\\ x_{21} &{}\quad x_{22} &{}\quad \ldots &{}\quad x_{1j} &{}\quad \ldots &{}\quad x_{2t}\\ \vdots &{}\quad \vdots &{}\quad \ddots &{}\quad \vdots &{}\quad \ddots &{}\quad \vdots \\ x_{i1} &{}\quad x_{i2} &{}\quad \ldots &{}\quad x_{ij} &{}\quad \ldots &{}\quad x_{it}\\ \vdots &{}\quad \vdots &{}\quad \ddots &{}\quad \vdots &{}\quad \ddots &{}\quad \vdots \\ x_{n1} &{}\quad x_{n2} &{}\quad \ldots &{}\quad x_{nj} &{}\quad \ldots &{}\quad x_{nt}\\ \end{array}\right) \end{aligned}$$

Source: The authors.

*Illustration 2. Average of volume index per region*

$$\begin{aligned} C=\left[ \begin{array}{l} c_1\\ \vdots \\ c_i\\ \vdots \\ c_n\\ \end{array}\right] \end{aligned}$$

*Illustration 3. Comparable volume index*

$$\begin{aligned} VC_{ij}=x_{ij} \times \frac{c_i/ \mathrm{Max}(c_n)}{\mathop {\sum }\nolimits _{n=1}^{n}x_{nj}/n} \times \frac{\mathop {\sum }\nolimits _{n=1}^{n}x_{it}/t}{\mathrm{Max}(c_n)} \end{aligned}$$

Source: The authors.

## Footnotes

1 The first paper analyses the lockdown of Wuhan (China) on 23 January 2020 and the second paper uses dummy variables to analyse different restrictions: localised recommended lockdown, localised lockdown, national recommended lockdown, national lockdown, workplace closure, stay-at-home orders, school and university closure, cancellation of public events, restriction of meetings, public transport closure, restriction of inland movement between cities/regions and restriction on inland travel.

2 Heterogeneity is shown in different fields, such as population concentration, economic activity, population structure, communication routes and decentralisation of policies, among others. In addition, other fields are important as the difference in weather factors or the decentralisation of healthcare (including the vaccination process), among others.

3 Both analyse the Wuhan (China) case and lockdown.

4 A reduction in pandemic severity means a decrease in the number of cases and deaths.

5 Pandemic severity is defined as the combination of new daily cases and deaths [3].

6 March and April were omitted due to a change in the data collection methodology by the Gabinete de Prensa del Ministerio de Sanidad, Consumo y Bienestar Social del Gobierno de España [52].

7 Although the primary data source is cited here, a dataset cleaned and standardised by Merelo [45], available on his github user, was used in the analyses.

8 The time period used has subsequently been shortened, due to the reduced time effect of this variable (noted above), to the period from 15 March 2020 to 31 October 2021.

9 Habitual mobility estimates the percentage of people who leave their area of mobility during working hours (10:00 AM to 4:00 PM) for more than 2 hours on the same day.

10 Area of residence is the area in which cell phones in Spain are located for the longest time between 00:01 AM and 06:00 AM for at least 60 days. The sample includes about 80% of the mobile market. The 3200 mobility areas identified are defined as population clusters of 5,000–50,000 inhabitants. In a depopulated area, the mobility area would be the sum of several small or very small municipalities (up to 5000 inhabitants). However, in cities, these areas could be districts or even parts of districts.

11 See Wooldridge [53, pp. 207–219].

12 See Wooldridge [54, pp. 403–404].

13 See Wooldridge [54, pp. 409–414].

14 This value is the result of adding the overall effect (3130) and the regional difference (1324).

15 A delay of 44 days is used due to the need for 30 days to achieve immunity and 14 days of incubation.
